# Feasibility of automated body trait determination using the SR4K time-of-flight camera in cow barns

**DOI:** 10.1186/2193-1801-3-225

**Published:** 2014-05-03

**Authors:** Jennifer Salau, Jan H Haas, Wolfgang Junge, Ulrike Bauer, Jan Harms, Sascha Bieletzki

**Affiliations:** Institute of Animal Breeding & Husbandry of Christian - Albrechts - University, Olshausenstraße 40, 24098 Kiel, Germany; Institute for Agricultural Engineering & Animal Husbandry of Bavarian State Research Center for Agriculture, Prof.-Dürrwaechter-Platz 2, 85586 Poing-Grub, Germany; GEA Farm Technologies, Siemensstraße 25-27, 59199 Bönen, Germany

**Keywords:** Dairy cow, Backfat thickness, Body condition, Automated monitoring, Time-of-flight, Image processing

## Abstract

As herd sizes have increased in the last decades, computerized monitoring solutions, which provide fast, objective and accurate evaluations of the herd status, gain more and more importance. This study analyzes the feasibility of a Time-of-Flight-camera-based system for gathering body traits in dairy cows for use under cow barn conditions. Recording, determination of body condition score on a 5 point scale by visual and manual inspection, and measuring the backfat thickness with ultrasound took place from July 2011 to May 2012 at the dairy research farm Karkendamm of the Institute of Animal Breeding and Husbandry, Christian-Albrechts-University Kiel (Germany) and between August 2010 and July 2012 at the Institute for Agricultural Engineering and Animal Husbandry of Bavarian State Research Center for Agriculture in Grub (Germany). The two breeds Holstein Friesian cows (Karkendamm) and Fleckvieh (Grub) were considered in this study. Software for recording, image sorting and evaluation, determining the body parts needed, and extracting traits from the images was written and assembled to an automated system. Sorting the images and finding ischeal tuberosities, base of the tail, and dishes of the rump, backbone, and hips had error rates of 0.2%, 1.5%, 0.1%, and 2.6%, respectively. 13 traits were extracted and compared to backfat thickness and body condition score as well as between breeds. All traits depend significantly on the animal and showed very large effect sizes. Coefficients of determination restricted to individual animals were reaching up to 0.89. The precision in measuring the traits and gathering backfat thickness was comparable. Results indicated that the application of Time-Of-Flight in determination of body traits is feasible.

## Background

### Body condition in dairy cows

During early lactation dairy cows experience negative energy balance which leads to more body tissue mobilization. As enduring negative energy balance strongly affects fertility performance and health, the body condition should be monitored systematically and accurately (Collard et al. 2000). At first, the body condition score (BCS) is a measure to describe a cow’s body condition. BCS is gathered by visually and manually judging the fat layer upon specific bone structures and how sunken the animal’s rear area is. There exist several systems using different scales. Thus BCS depends on the person who classifies. In this study the 5 point scale system developed in (Wildman et al. 1982) and (Edmundson et al. 1989) is used. At second, the layer of subcutaneous fat which is bounded by skin and the fascia trunci profunda located at the gluteus medius muscle, respectively the longissimus dorsi muscle (Schröder and Staufenbiel 2006) is called backfat. Measuring the backfat thickness (BFT) with ultrasound is highly reflective of the body fat content and highly correlated to BCS (Brethour 1992; Fietze 2004). However, this method is very time consuming, because the animals have to be fixed, and the measurements depend on the used technical equipment. The body condition’s use as monitoring tool for diet, health, and fertility status would increase, if it could be obtained objectively and automatically. Additionally, interest in the body condition as selection index rises (Interbull 2012). An automatic system would lower costs and the expenditure of time, be less stressful for the animals, avoid errors during manual data transcription, and could provide large volumes of data for use in genetic evaluation. The present study therefore examines the usage of Time-Of-Flight (TOF) depth cameras in automated monitoring of the body condition in lactating dairy cattle.

### Usage of camera-based systems in dairy precision farming

Technological advances and multi-disciplinary research will be essential tools in the 21st century’s agricultural science. Digitally stored results can easily serve as basis for further research and data mining. In the last years, there have been several 2D-camera-based studies on automated body condition scoring. After the potential of the use of 2D digital images in BCS determination was demonstrated (Bewley et al. 2008; Ferguson et al. 2006), various approaches to automated systems were made. In (Azzaro et al. 2011) cow shapes were reconstructed using linear and polynomial kernel principal component analysis and the BCS was estimated. BCS prediction models based on five anatomical points were presented in (Bercovich et al. 2012). Full automation was reached in (Halachmi et al. 2013), BCS was assessed by fitting a parabola to the cow shape extracted from thermal images. This study analyzes the applicability of a Time-of-Flight (TOF) 3D camera in automated determination of cows’ body traits (Salau et al. 2011). With 2D coordinates, everything can only be measured in a certain plane of projection depending on the cameras point of view. The ability to measure spatial anomalies is, however, necessary to fully describe a three dimensional object. As in 3D images the pixels’ relative distances from the camera are known, the separation between fore- and background can be achieved easier than the segmentation of 2D digital images (Hertem et al. 2013). In (Krukowski 2009) images from the previous model of the TOF camera used in the present study were analyzed. Dairy cows’ rear view was captured with the camera held in hand, and a relatively small number of animals and images were examined in order to determine BCS. Automation was not aspired. The present study introduces a TOF-based system with automated calibration, animal identification and information gathering. To cover a wider range of body shapes and sizes, two breeds (Holstein Friesian (HF) and Fleckvieh (FV)) were recorded. The same traits were calculated for both breeds and afterwards compared to BFT and BCS.

## Results

A rough program flow of the whole software is given at the beginning of subsection "Developed software". In paragraphs "Configuration and recording" to "Further processing: application "ExT"" the single processing steps are explained in more detail. Error rates and technical data are given in "Analyzing the software’s results".

### Developed software

**Figure 1:**
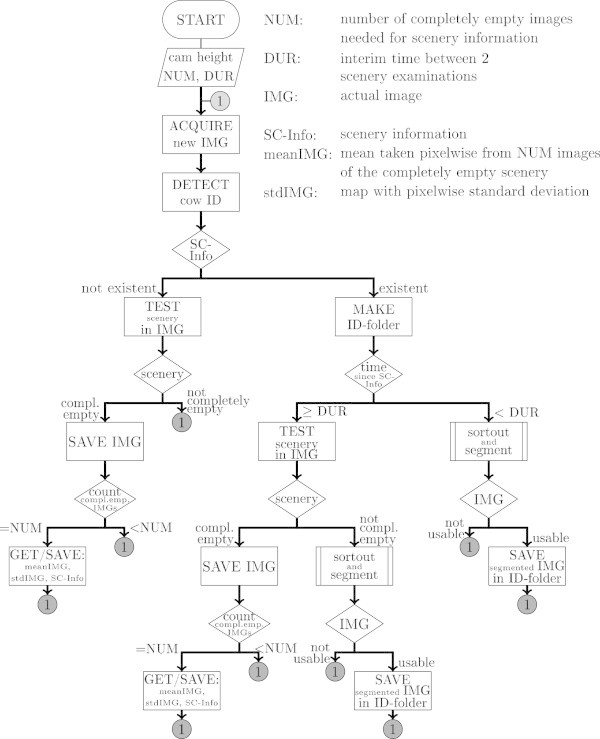
**Flow chart of "Karkendamm.exe".** Using the parameters given in the configuration file, "Karkendamm.exe" set up the camera, connected to the ID-system, and initiated calibration. After scenery-information had been determined the acquired images were sorted, segmented, and stored in ID-folders. Termination criteria are not shown for the sake of simplicity.

Initially, the application "Karkendamm.exe" (Figure 1, ((FNI) FI 2013), (Crémer 2013)) reads configuration files, where camera settings and recording parameters were specified, and started animal identification, calibration, recording, and preprocessing.

**Figure 2:**
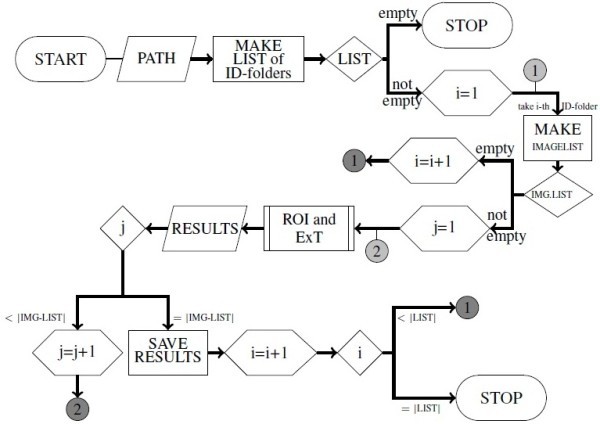
**Flow chart of "EKB".** The application "EKB" controlled further processing. It handed every image to the applications "ROI" and "ExT" (automated determination of region of interest and trait extraction) and listed as well as stored the output.

**Figure 3:**
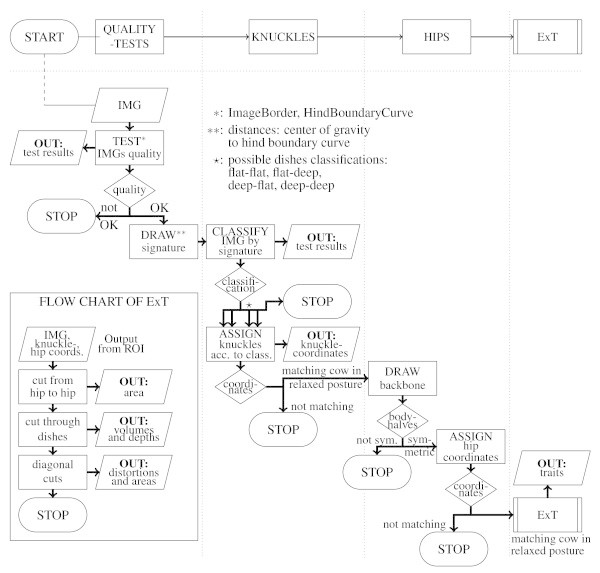
**Flow chart of "ROI" and "ExT".** "ROI" evaluated IMG in a multistage system of tests (ImageBorder and HindBoundaryCurve, first column). It classified the signature of the cow’s backside contour, determined ischeal tuberosities, base of the tail, dishes of the rump and tested their coordinates for anatomical correctness (second column). Backbone and hips were also determined and tested automatically (third column), before the image was handed to "ExT" (fourth column). "ExT" calculated the camera traits (separated box).

Afterwards the MATLAB application "EKB" controlled the further processing (Figure 2). "EKB" handed every image to the MATLAB applications "ROI" and "ExT" (Figure 3), in order to automatically determine the region of interest and to extract traits, respectively. In addition, "EKB" generated one list per recording day, that for every valid image contained the output of "ROI" and "ExT".

As this study focuses on the feasibility of a TOF based automated monitoring system, prediction of neither BFT nor BCS was integrated in the software so far.

#### Configuration and recording

In the configuration files the user could determine calibration parameters (NUM, camera height, DUR, see paragraph "Calibration and preprocessing"), what kind of output (subsection "Time-of-flight technology") should be saved and where, and if the images should be preprocessed during recording. Additionally, the user could predefine a time or amount of data at which the recording terminated. After this configuration the camera was set up, and the application connected to the ID-system used at Karkendamm. Then acquisition of images continued until the termination conditions were fulfilled.

#### Calibration and preprocessing

"Karkendamm.exe" used the camera height to determine, if the scenery was completely empty. From a specified number (NUM) of such images scenery information (SC-Info) were calculated. Firstly, this included maps with pixelwise mean (meanIMG) and standard deviation (stdIMG) of the empty scenery. Secondly, examining edges and gradients in meanIMG resulted in the position of box walls. After a specified duration (DUR) SC-Info was renewed.

**Figure 4:**
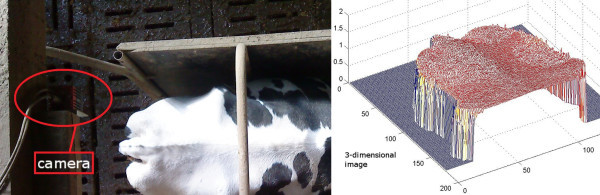
**Scenery, recording installation, and example of preprocessed TOF camera output.** **Left:** TOF camera mounted above an electronic feeding dispenser in Karkendamm; **Right:** Segmented 3D-representation of a cow’s rear area.

Given SC-Info, "Karkendamm.exe" created a folder (ID-folder) named after the cow-ID linked to the actual image (IMG). As the camera was recording nonstop, the cows were observed while advancing into and moving backwards out of the box again. As only images showing a cow’s lower back should be stored in ID-folder for further processing, "Karkendamm.exe" handed IMG to the application "sortout_and_segment". Comparing the depth values in IMG to the specified camera height, "sortout_and_segment" determined whether there was an object between the box walls and where. Due to the mounting of the camera (Figure 4), and because the cows could only enter the box from one side, they were passing the camera’s field of view from the upper to the lower image boundary. If IMG showed a lower back including the tail, the object necessarily had to intersect with the lower image boundary and to keep distance to the upper image boundary. If that was not the case, IMG was deleted automatically to reduce data volume. Otherwise "sortout_and_segment" separated the cow from the background. Hereby the difference |IMG-meanIMG| was used to specify moving objects at first. At second, objects were recognized by means of depth value histograms, to confirm the first result. The background was set to zero, and the segmented IMG was stored in ID-folder afterwards.

This resulted in one ID-folder of preprocessed images per day for every cow, which had been in the electronic feeding dispenser while recording. Multiple visits of the same animal were stored in the same ID-folder.

#### Further processing: application "ROI"

**Figure 5:**
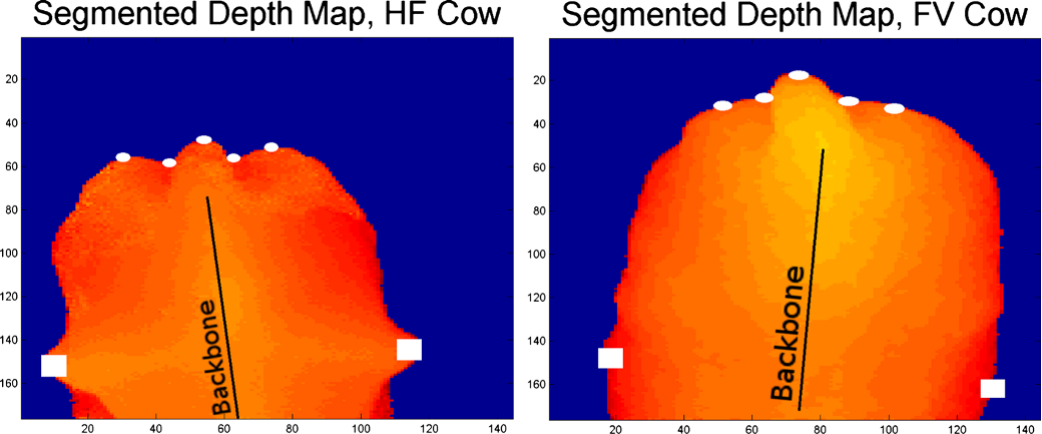
**Determining the region of interest.** The left respectively the right figure shows a depth maps of a HF and a FV cow’s backside recorded from top view. Everything but the cow is set to zero (blue). The automatically determined region of interest is marked: White dots: Ischeal tuberosities, dishes of the rump and tail (knuckles). White rectangles: hips.

The application "ROI" evaluated IMG in a multistage system of tests and determined body parts that are important descriptors for body condition scoring like ischeal tuberosities, base of the tail, dishes of the rump, hips, and backbone (Figure 5, (Ferguson et al. 1994)).

#### Evaluating the image: tests ImageBorder and HindBoundaryCurve

**Figure 6:**
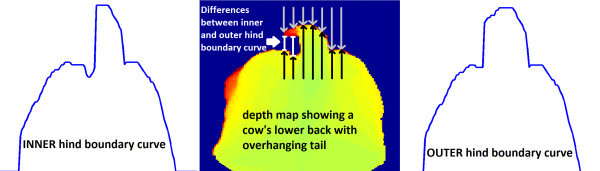
**Calculating and comparing the inner and outer hind boundary curves.** **Mid:** Depth map of cow’s backside recorded from top view. Everything but the cow is set to zero (blue). **Left/Right:** The inner/outer hind boundary curve. The inner/outer hind boundary curve is calculated by following the columnwise black/grey arrows to the last/first nonzero pixel. As the cow’s tail is overhanging, the inner and outer hind boundary curve differ (marked as white lines).

The first test (test ImageBorder) checked, if the cow sticked to the left or right image border. At second (test HindBoundaryCurve), the hind boundary curve was determined in two ways: as sequence of first nonzero pixels from the upper image boundary in direction of the segmented object and as sequence of last nonzero pixels from within the object in direction of the upper image boundary (Figure 6). Objects (pieces of wall, floor or other cows), which had not been removed by "sortout_and_segment" produced differences between these two hind boundary curves.

#### Determination of the region of interest

**Figure 7:**
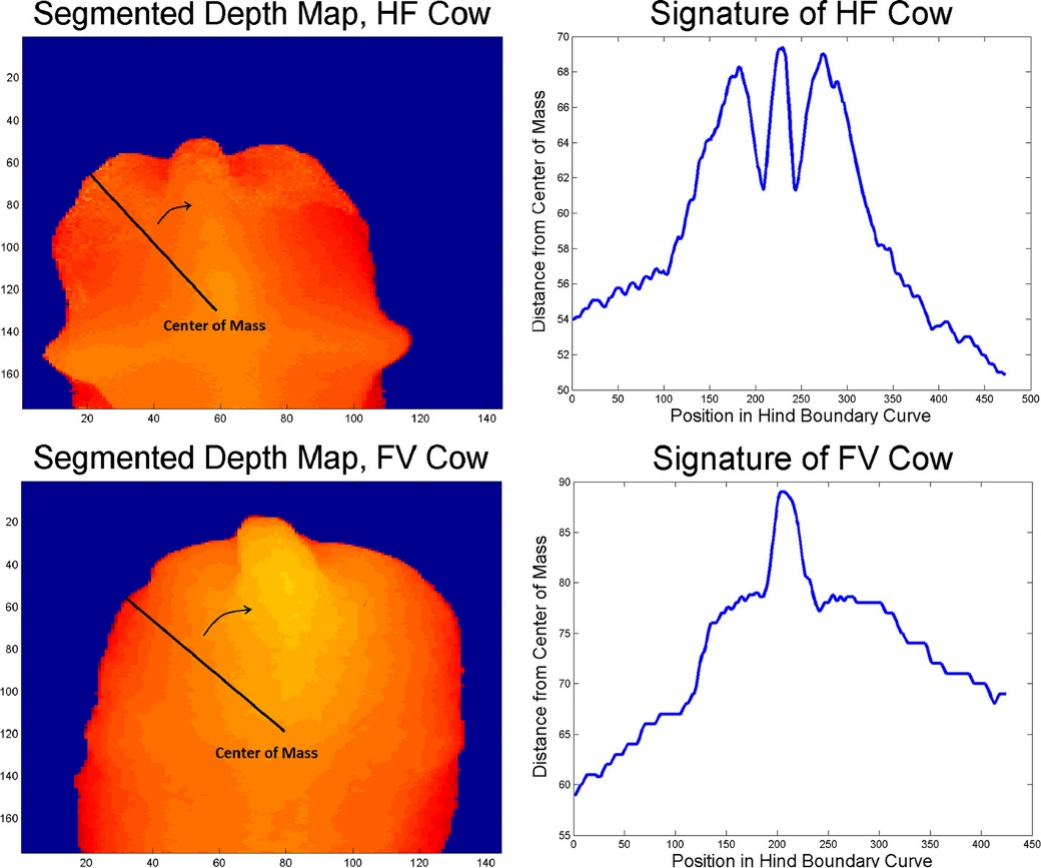
**Generating signatures and comparing between the breeds HF and FV cows.** The left column of images shows depth maps of a HF (top) and a FV (bottom) cow’s backside recorded from top view. Everything but the cow is set to zero (blue). In the right column the signatures (top: HF cow, bottom: FV cow) generated from the corresponding depth maps in the left column are displayed. In both depth maps the center of mass calculated for the area covered by cow (nonzero) is marked. The signatures are generated by measuring the distances between center and boundary of the nonzero area. As the breed’s pelvis shapes differ and ischeal tuberosities lie more rearwards relative to the center of mass in HF than in FV cows, signatures of HF and FV cows are clearly distinguishable.

If IMG had passed those tests, the signature of the cow’s backside (also used in (Bercovich et al. 2012)) was drawn by calculating the distances between every point of the hind boundary curve and the center of mass. It could be observed that, the distances between ischeal tuberosities and center as well as tail and center are similar in HF cows, but in FV cows the tail is considerably the farthest point measured from the center. These differences in pelvis shapes resulted in dissimilar signatures (Figure 7). As ischeal tuberosities, dishes of the rump and tail (all together referred to as knuckles) were detected from the signature, slightly different algorithms were used in their detection for the two breeds:

For both breeds the tail was detected as the signature’s local maximum in alignment with the center of mass.

**Figure 8:**
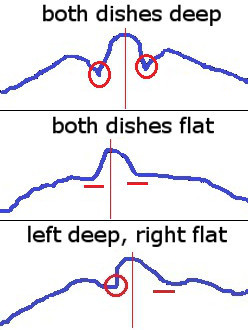
**Three examples of signatures.** The tail is marked with a vertical red line in all three images. **Top:** Minima are easily detectable on both sides of the tail bump. **Mid:** The signature segues from tail bump into a flat course. **Bottom:** A minimum can be detected left of the tail, but the right dish is flat.

The animal’s individual anatomy but also actual posture caused the dishes of the rump to appear flat or deep in the signature. If an explicit local minimum could be found sideways of the local maximum corresponding to the cow’s tail, the dish of the rump was detected there and classified deep (Figure 8, top). It was classified flat, if the signature’s gradient was running flat to the side (Figure 8, mid). The dish was then detected at the bottom of the tail’s associated bump in the signature where the curvature was largest.

It indicated equally stressed hind legs and the tail in a mid-position, when both dishes got the same classification. In HF cows only images with both dishes classified as deep were used. The ischeal tuberosities were detected as the signature’s local maxima further left than the left dish and further right than the right dish. As in FV cows flat dishes were outweighing, also images with both dishes classified as flat were used. For those images the points, where the signature decreased below a specified tolerance, were detected. The ischeal tuberosities were set halfway between the dishes of the rump and those points.

As a next step, a linear approximation of the highest points on the cow’s back provided the backbone. This gave the opportunity to compare the body halves and assign hip coordinates in case they were sufficiently symmetric.

To confirm the assumption, that the object analyzed in IMG was a cow in relaxed posture, lengths and angles within the signature as well as the knuckle and hip coordinates determined from left and right were compared. Both their distances to the tail, respectively backbone, and their depth values had to be about the same size. They were discarded otherwise.

#### Deciding which algorithm to use

The breed (HF - or FV cow) had to be given to the application "ROI" as input argument. It would be possible to automate this flow of information concerning the cow’s breed given a mapping between ‘breed’ and ‘ID’ that is accessable from the herd management system. In this way the breed of every recorded cow could have been available, because the software was connected to the ID system. As there was no opportunity to test the system in herds with both breeds, this step has not been carried out yet.

#### Further processing: application "ExT"

**Figure 9:**
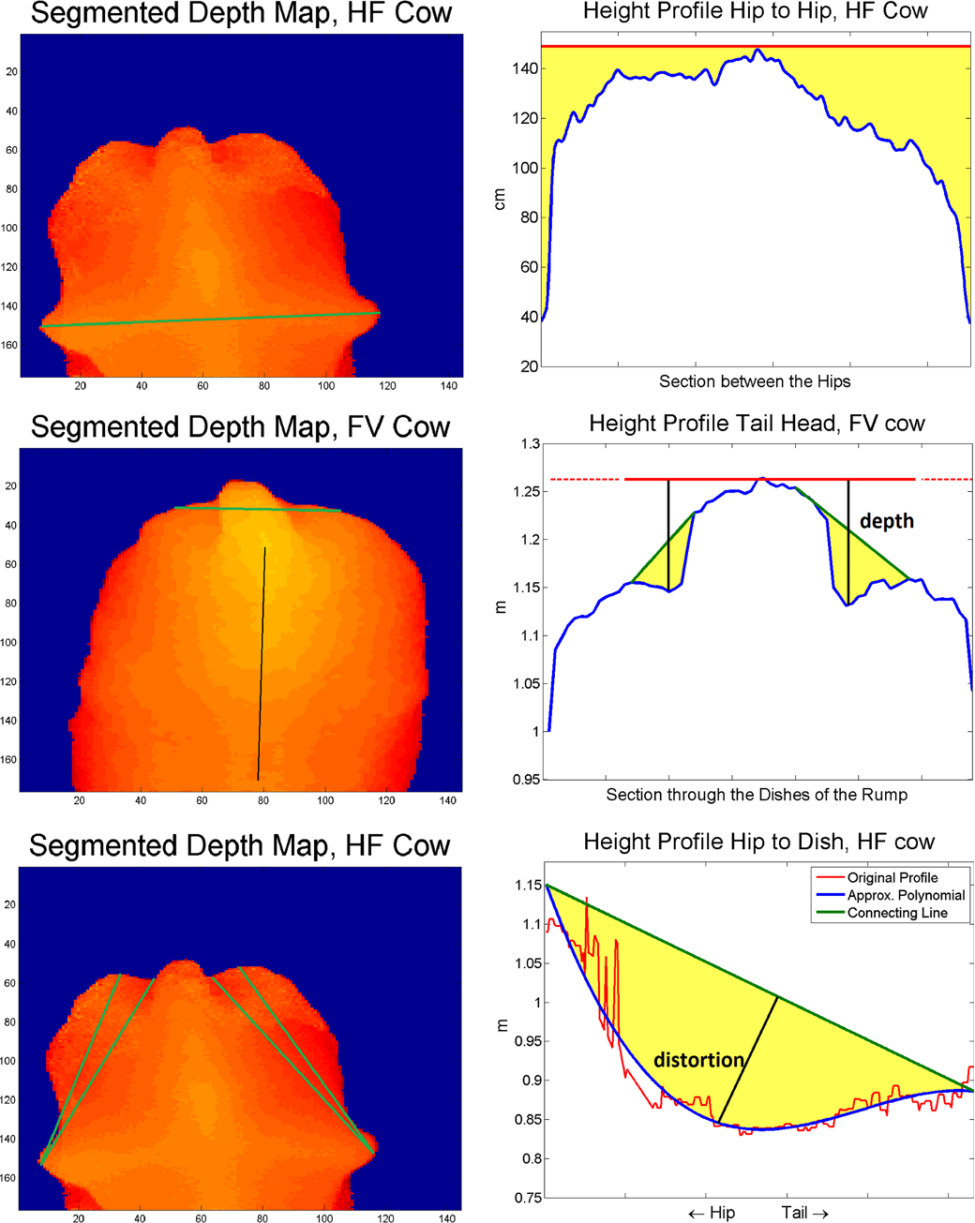
**Graphic representation of the camera traits.** **Top, left:** Connecting line hip to hip. **Top, right:** The area between cow profile and horizontal tangent gives the trait hip2hip. **Mid, left:** Cutting line through dishes of rump. **Mid, right:** Cow profile, horizontal tangent, closing lines. The depth of dishes and the area between profile and closing lines are calculated for the traits depthleft/-right and volumeleft/-right. **Bottom, left:** Connecting lines hips to ischeal tuberosities and dishes. **Bottom, right:** Cow profile, polynomial approximation, spatial connecting line. Calculating the area gives the traits areapinleft/-right and areamidleft/-right. The traits distpinleft/-right and distmidleft/-right are calculated as the maximal distortion between the polynomial and the connecting line.

In case the complete region of interest could be determined, IMG was passed on to "ExT". Several cuts through the cow’s surface were taken along straight lines in order to calculate traits that are meaningful to the surface’s changes induced by varying body condition during lactation: As a first trait (Figure 9, top), the area between the height profile along the connection between the cow’s hips and its upper horizontal tangent line was computed (hip2hip). At second, a cutting line was drawn perpendicularly to the backbone near by the dishes of the rump (Figure 9, mid). Using the resulting height profile, the depths of the dishes (depthleft, depthright) and the areas between the profile and the closing lines (volumeleft, volumeright) were measured. At last, height profiles were taken between hips and ischeal tuberosities or dishes of the rump on both sides of the body. Their polynomial approximation was compared to the direct spatial connecting line. The maximal distortions (distpinleft, -right, distmidleft, -right) as well as the included areas (areapinleft, -right, areamidleft, -right) were calculated (Figure 9, bottom). Per valid image 13 traits (subsequently referred to as camera traits) could be computed.

### Analyzing the software’s results

#### Technical information on recording

Used with default settings and under the given light conditions, the SR4000 recorded ≈14 frames per second (fps) without carrying out "sortout_and_segment", and ≈3 fps otherwise. After 1st of November 2011 only Swiss Ranger streams (srs) each containing 1000 frames were recorded and used as data source afterwards. It took ≈70 seconds to record one stream of size 99065 kb. This summed up to ≈5.1 gb recorded srs data per hour. Using streams as data source, single images could be gathered and preprocessed by "sortout_and_segment" with ≈35 fps depending on the computer’s performance.

#### Control of preprocessing and determination of ROI

All images used for visual inspection were recorded with the exact same settings as described in subsection "Installation and recording" but prior to the recording period used for analysis (specified in section "Methods").

The results of "sortout_and_segment" (paragraph "Calibration and preprocessing") were controlled visually at a dataset of 155322 images from recording days 8th and 9th of June 2010 containing 133 different cows. The error rate was 0.2%, and ≈36% of all images were kept. The combined results of the tests ImageBorder and HindBoundaryCurve (paragraph "Further processing: application "ROI"") were visually tested by scientific assistants using the recording of 13th of July 2011, which contained 215085 images from 84 different cows. Distribution, absolute number of images, and the error rates are given in Table 1. Knuckle coordinates were only calculated from the 192798 images which had passed both ImageBorder and HindBoundaryCurve, and the coordinates were evaluated. The positively evaluated knuckle coordinates (163490 images ≈ 85%) were visually checked. The error rate was 1.5%. Furthermore, 39836 images were randomly chosen from the recording dates between 5th of July and 18th of August 2010 and used for visual inspection of the automatically determined backbones (error rate: 0.1%) and hip coordinates. The positively evaluated hip coordinates (25893 images, ≈ 65%) had been assigned with 2.6% error rate.

**Table 1:** **Distributions, absolute numbers of images, and error rates of the tests ImageBorder and HindBoundaryCurve**

Code	%	Absolute	Error rate	Use
1-1-1	89.6	192,798	20.6%	further processing
1-0-1	2.7	5,856	5.9%	discarded
0-1-1	6.7	14,493	13.4%	discarded
0-0-1	1	1,940	22.3%	discarded

The results are coded as triples of 0 (failed test) or 1 (passed test). First entry: HindBoundaryCurve; second/third entry: ImageBorder left/right side. Groups "1-1-0", "1-0-0", "0-1-0", "0-0-0" are empty.

#### Traits’ descriptive statistics and coefficients of determination

Table 2 gives the descriptive statistics for all traits for Karkendamm and Grub, separately. The coefficients of determination for individual cows  (explained in subsection "Descriptive statistics") were not significantly effected by the factors weight, lactation number, lactation stage at the beginning of the recording period (compare subsection "Additional information on the recorded dairy cows"), or BFT/BCS starting level. As no meaningful criterion was found to limit the dataset to certain animals, all cows’ data was used for analysis. For HF cows the coefficients of determination *R*^2^ ranged from 0.7 to 0.93 for the camera traits and *R*^2^ of BFT was 0.86 (mean(*R*^2^) = 0.83, ±0.07). The mean and maximal -value for BFT were 0.43 and 0.96, respectively. For the camera traits mean -values between 0.17 and 0.28 and maximal -values between 0.59 and 0.89 were observed.

**Table 2:** **Descriptive statistics of all traits for Karkendamm and Grub data set**

KARKENDAMM								
Trait	N	min	max	mean	std	***R*** ^2^ ( mean,max)	corrBFT	-
BFT	2848	5	28	9.71	3.78	0.86 (0.43,0.96)	1	-
HH	1946	2.4	135.7	38.21	12.83	0.87 (0.21,0.78)	-0.16	-
DL	1979	1.3	32.1	12.20	2.66	0.86 (0.26,0.84)	-0.23	-
DR	1984	2.7	28.1	12.44	2.67	0.88 (0.28,0.81)	-0.25	-
VL	1933	0.8	58.1	13.59	5.63	0.67 (0.24,0.89)	-0.19	-
VR	1937	0.7	54.4	13.57	5.41	0.78 (0.21,0.69)	-0.15	-
DPL	1920	0.5	15.5	5.37	2.13	0.89 (0.22,0.84)	NS	-
APL	1904	0.4	37.6	7.37	4.22	0.81 (0.17,0.59)	0.11	-
DML	1915	0.3	8.9	3.71	1.43	0.93 (0.25,0.89)	0.22	-
AML	1910	0.4	23.1	6.77	3.52	0.88 (0.24,0.74)	0.26	-
DPR	1906	0.6	20.0	6.14	3.12	0.82 (0.2,0.74)	NS	-
APR	1903	0.2	42.3	9.39	6.29	0.7 (0.17,0.69)	NS	-
DMR	1910	0.2	13.8	4.35	2.23	0.85 (0.24,0.89)	0.08	-
AMR	1918	0.2	32.9	8.65	5.47	0.76 (0.22,0.79)	NS	-
**GRUB**								
**Trait**	**N**	**min**	**max**	**mean**	**std**	***R*** ^**2**^ **(** **mean,max)**	**corrBFT**	**corrBCS**
BCS	540	2.5	5	3.76	0.43	0.7 (0.25,0.66)	0.41	1
BFT	542	9	46	20.42	5.88	0.49 (0.24,0.63)	1	0.41
HH	514	22.9	62.3	37.12	6.3	0.59 (0.14,0.67)	NS	0.31
DL	514	7.2	27.6	12.90	2.39	0.71 (0.23,0.95)	-0.15	-0.3
DR	514	6.6	21.4	12.71	2.93	0.83 (0.26,0.86)	-0.32	-0.46
VL	514	0.5	50.9	5.55	4.0	0.43 (0.18,0.84)	-0.06	-0.14
VR	514	1.1	24.2	5.86	3.67	0.63 (0.21,0.83)	-0.27	-0.38
DPL	514	0.2	3.6	1.37	0.51	0.53 (0.19,0.69)	NS	NS
APL	514	0.2	5.6	2.11	0.95	0.52 (0.2,0.6)	NS	NS
DML	514	0.3	4.8	1.43	0.62	0.55 (0.21,0.66)	-0.07	NS
AML	514	0.4	14.5	2.66	1.6	0.6 (0.23,0.83)	-0.07	-0.07
DPR	514	0.2	5.8	1.27	0.55	0.34 (0.14,0.52)	NS	NS
APR	514	0.1	5.9	1.34	0.74	0.41 (0.14,0.39)	NS	NS
DMR	514	0.5	10.1	1.76	1.22	0.55 (0.16,0.54)	-0.18	-0.15
AMR	514	0.6	22.5	2.87	2.96	0.47 (0.18,0.67)	-0.14	-0.12

The traits were specified in column 1 using the following abbreviations: BFT – backfat thickness, BCS – body condition score, HH – hip2hip, DL – depthleft, DR – depthright, VL – volumeleft, VR – volumeright, DPL – distpinleft, APL – areapinleft, DML – distmidleft, AML – areamidleft, DPR – distpinright, APR – areapinright, DMR – distmidright, AMR – areamidright (compare List of Abbreviations and camera trait description in paragraph "Further processing: application "ExT"" and Figure 9). Coefficients of determination had been calculated restricted to individual cows (*R* ^2^_*cow*_, mean and maximum in brackets, all minimums zero) and for the whole datasets using a generalized linear model with a piecewise linear link function (*R* ^2^). Last columns contain the correlations to BFT (corrBFT) and BCS (corrBCS), *p* = 0.05, "NS" indicates that the linear connection was not significant.

For FV cows minimal and maximal *R*^2^-values were 0.34 and 0.83 for camera traits, *R*^2^ of BCS and BFT were 0.7 and 0.49, respectively (mean(*R*^2^) = 0.56, ±0.13). Mean -values ranged between 0.14 and 0.26 and maximal -values between 0.52 and 0.95 for the camera traits. BCS and BFT had mean -values 0.25 and 0.24 and maximal -values 0.66 and 0.63, respectively.

Minimal -values were zero for all camera traits and BFT/BCS for both breeds.

#### Effects of cow and season

The sizes (*η*^2^) for both cow and season effects are presented in Table 3. For both breeds the cow’s effect on the group means was significant and very large (Cohen 1988), ranging from *η*^2^ = 0.59 to *η*^2^ = 0.8 for HF cows and from *η*^2^ = 0.34 to *η*^2^ = 0.77 for FV cows. Means grouped after season only differed significantly for BFT and the camera traits depthleft, depthright, distpinleft, areapinleft, distmidleft, and areamidleft for HF cows. For FV cows significant differences could be observed for BFT, hip2hip, depthleft, volumeleft, volumeright, areapinleft, areamidleft and distpinright. The effect sizes were small (*η*^2^ = 0.01 to *η*^2^ = 0.03) for HF cows. For FV cows the sizes were small to medium (*η*^2^ = 0.02 to *η*^2^ = 0.06) for the most traits, but for volumeleft the season’s effect could almost be considered large (*η*^2^ = 0.12).

**Figure 10:**
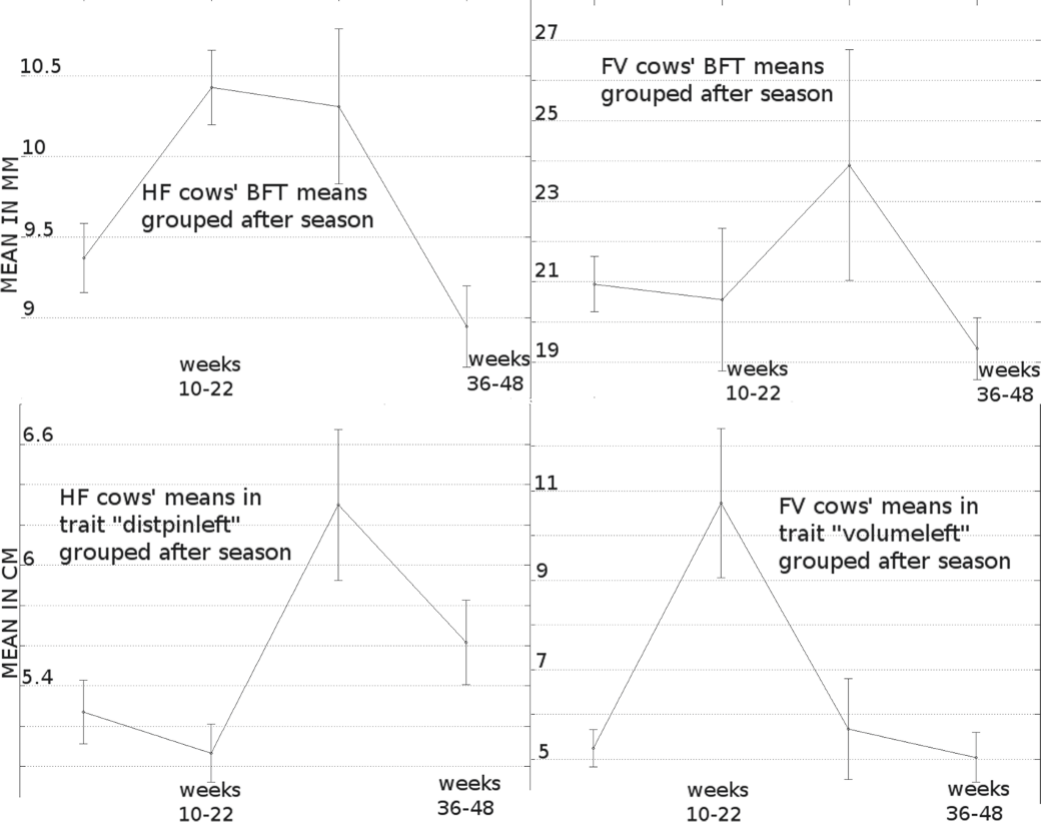
**Group means for data grouped after season.** The year has been subdivided in the following seasons: season 1: weeks 49-52, 1-9; season 2: weeks 10-22; season 3: weeks 23-35; season 4: weeks 36-48. Group means for BFT (top) and the camera traits with maximal effect size for HF (left, trait distpinleft) and FV (right, trait volumeleft) cows are presented. Non intersecting confidence intervals indicate significant differences in group means.

Figure 10 illustrates the season means for BFT for both breeds as well as for distpinleft for HF cows and volumeleft for FV cows, as for these traits the sizes of the season’s effect were maximal.

**Table 3:** **Effect sizes (** ***η*** ^***2***^ **) cow and season effect**

	Size of cow effect		Size of season effect	
Trait	Karkendamm	Grub	Karkendamm	Grub
BCS	-	0.6	-	NS
BFT	0.59	0.39	0.02	0.04
HH	0.65	0.53	NS	0.06
DL	0.69	0.64	0.02	0.06
DR	0.71	0.77	0.02	NS
VL	0.79	0.34	NS	0.12
VR	0.76	0.56	0.01	0.04
DPL	0.71	0.43	0.03	NS
APL	0.70	0.42	0.01	0.02
DML	0.75	0.49	0.02	NS
AML	0.76	0.54	0.01	0.02
DPR	0.75	0.29	NS	0.02
APR	0.75	0.34	NS	NS
DMR	0.78	0.51	NS	NS
AMR	0.8	0.43	NS	NS

The traits were specified in column 1 using the following abbreviations: *BFT* backfat thickness, *BCS* body condition score, *HH* hip2hip, *DL* depthleft, *DR* depthright, *VL* volumeleft, *VR* volumeright, *DPL* distpinleft, *APL* areapinleft, *DML* distmidleft, *AML* areamidleft, *DPR* distpinright, *APR* areapinright, *DMR* distmidright, *AMR* areamidright (compare List of Abbreviations and camera trait description in paragraph "Further processing: application "ExT"" and Figure 9). "NS" indicates that group means did not differ significantly. Means between cows were significantly different with very large effect sizes for all traits. Season effects were small to medium, except for "VL" within the Grub dataset.

## Discussion

### Technical aspects of the presented system

The TOF-based system presented in this study was able to carry out the tasks camera setup, calibration, animal identification, image acquisition, sorting, segmentation, and the determination of the region of interest as well as the extraction of body traits automatically. "Karkendamm.exe" handed folders with preprocessed images to the MATLAB application "EKB" to control further processing and store the results (subsection "Developed software"). The image processing steps, however, turned out to be very time consuming, therefore the presented software was not a real-time application. When preprocessing was carried out while the camera was recording, its acquisition rate dropped from ≈14 to ≈3 fps. After 1st of November 2011 thus only Swiss Ranger streams of unprocessed images were recorded. Afterwards, the serial execution of "Karkendamm.exe" and "EKB" was carried out using srs data as virtual camera. Since the amount of data was maximized (fps, recorded cows) by keeping a strict separation of recording and processing, this was considered the best solution for a serialized scenario. Parallel solutions were thinkable using connected hardware systems, and they would be necessary if the goal is real-time recording and processing.

### Camera trait extraction

The application "sortout_and_segment" guaranteed a perfect sorting (0.2% error rate) of the images depending on if they showed the rear area of a cow.

Because the position of the freely movable tail was difficult to judge automatically, the tests ImageBorder and HindBoundaryCurve showed relatively high error rates. Furthermore abrupt changes in the distance between recorded object and camera led to more possible ways for the infrared light to be reflected and return to the sensor. Automatically and precisely deciding between cow and a wall very closed to the cow was prone to error, because the strong slope between cow surface and floor led to increased depth value deviations. But as the knuckle and hip coordinates were evaluated with 1.5% and 2.6% error rate, respectively, a very reliable basis for trait extraction was laid.

However, for both breeds the traits’ standard deviations and the ranges in values were high. One reason for this might be the quality of depth measurement. According to the manual (MESA-Imaging 2013) the accuracy was 1 cm, but depth value deviation of up to 10 cm had been observed. As preliminary analyses had shown, this most likely depended on fur color (changes), sun light, dust, insects, humidity, and if the animal was moving. The effects of these parameters, however, could so far not be separated or proven to be significant.

Other reasons might lay in differences between the animals. As was shown, the cow had a significant effect on all traits. Individual body shapes, fur colors, and changes in fat deposits might have been reasons for this as well as the cows’ behavior during recording which affected image quality and ROI determination (compare subsection "Comparing measurement precision").

Having a better control over cows, recording and scenery, the image quality might improve. A solution would have been to build an artificial scenery, that ensured symmetry and optimal conditions for the camera. The advantage would be better control over light, animal movement, dust, insects, and humidity. Also parallel image processing and recording could have been facilitated and, in turn, computation time could have been reduced. But as costs and efforts would increase, and in some existing cow barns such an installation might not be possible, the applicability and versatility as an everyday use camera-based monitoring system would be limited.

#### Correlations to BCS/BFT within breeds, potential BCS/BFT estimation

The angles and distances extracted in (Bercovich et al. 2012) could easily be calculated from the signature (paragraph "Further processing: application "ROI"", Figure 7), and also a parabola as used in (Halachmi et al. 2013) could be fitted to the cow’s shape in the segmented images. The corresponding procedures in BCS determination had in literature been proven to work well and had therefore not been implemented in the TOF based system presented in this study.

Instead, the TOF camera’s ability to provide depth data was used to gather additional information on the cow’s surface, and the extracted traits were analyzed according to their explanatory power and precision in determination.

As none of the camera traits could fully describe the individual changes in fat layer or body condition by itself, the correlations to BFT were low or not significant. An additional explanation was, that none of the traits dealt with the exact BFT measurement point, which would have been difficult to detect from the TOF recordings. The significant correlations to BFT occurred mainly with traits dealing with the tailhead region, but irregularly also with the diagonal sections between the hips and the tail region and the area under the hip to hip connection. For inferential statistics aiming to predict BFT from the presented camera traits, it could be promising to consider a set of predictors that delivered information from the whole surrounding area of the BFT measurement point (Weber et al. 2014).

The correlations between camera traits and BCS (only FV cows) were moderate for all traits except the ones calculated from the diagonal sections. Confirming (Ferguson et al. 1994) the camera traits extracted from the tailhead region were most promising in a further BCS prediction.

#### Seasons effects

Taking into account that e.g. the number of flying insects, sun light, or fur structure underlay seasonal variations and effected the image quality, significant differences in the traits’ means among seasons were to be expected. These effects were medium to large in the recording of FV cows in Grub, but not for all traits significant. For the recording of HF cows in Karkendamm the seasonal effects were small. It was, however, noticeable that only all traits extracted from the left side and exceptionally the trait depthright were effected significantly. This might have been caused by asymmetries in recording scenery. The camera was positioned accurately above the box’s longitudinal axis, but the box’s walls were not equally made, and the recording scenery was exposed to different influences from left and from right (description in subsection "Installation and recording"). Additionally, depending on the walls’ materials the cows might have had preferences in leaning on one wall more often than the other.

### Comparing measurement precision

#### Overall coefficients of determination

For both breeds and all camera traits as well as BFT/BCS the same generalized linear model with a piecewise linear link function was used to compute coefficients of determination. The purpose had not been prediction, but providing a measure to compare the precision of methods between manually gathering BFT/BCS and calculating traits with the presented software.

In HF cows BFT and camera traits had been measured comparably precise. Therefore, it could be promising to establish a larger dataset for the camera traits and to analyze their usability in functional traits.

In FV cows the *R*^2^ - values were lower and their range was wider. As the groups in this study happend to be unbalanced the significance of the breed’s effect on *R*^2^ - values has not been analyzed. Within the FV dataset, most of the camera traits showed a comparable *R*^2^ to that of BFT. For BCS a considerable higher coefficient of determination was computed than for BFT. The precision in BCS exceeded the precision in measuring most of the camera traits. Exceptions were the depths of the dishes of the rump. Although BFT gathering also included touching the animal, BCS is the only trait considered in this study which involved palpation of the fat layers. This might indicate that purely visual determination of body traits or detecting the measurement point for the ultrasound device could be more difficult with FV cows than HF cows. As the coefficients of determination were still moderate to high, a larger dataset to analyze a potential usage in functional traits should be considered.

#### Individual coefficients of determination

The wide spread between the -values showed, that some cows abetted measuring both camera traits and BFT/BCS, and from other cows hardly any reliable measurements could be gathered. But similarity of cows was difficult to quantify because of the high number of comparative aspects. The superficial reasons cow height, weight, lactation, BFT/BCS starting level, and lactation stage had been excluded as significant influences (paragraph "Traits’ descriptive statistics and coefficients of determination"). Further careful analyses have to be done here, to find meaningful criteria for cows’ measurability and to explain the trait values’ deviation in more detail.

#### Observed differences between breeds

FV cows had lower maximal  - values and means, indicating a reduced reliability in information gathering in comparison to HF cows. FV cows had less visible bone structure in the concerning area, and their body shapes showed greater convexity, i.e. the surface was less sunken between the bones. As a result, the extracted profiles expressed less distortion from a spatial connecting line or described smaller areas. The camera traits were thus more susceptible to deviation in depth measurement as well as to smoothing and rounding values. The FV cows’ convex body shape could also be recognized in smaller max-min differences for the camera traits dealing with diagonal sections through the rear area or the area under the hip to hip connection compared to HF cows.

The dissimilar group sizes have to be named as an other potential reason for the observed differences. As the Karkendamm herd had free access to the electronic feeding dispenser equipped with the camera, no influence could be taken in advance which and how many animals were recorded. Because the groups were unbalanced by factor three, no analysis of variance has been done with the breed as factor. The present study discovered a large cow effect regarding all traits, and no significant characteristic to group the cows and explain the differences in individual measurability could be found. The effect of balancing the groups afterwards could not have been controlled.

## Conclusion

The whole process of automated information gathering had been implemented, and since traits could be gathered at comparable precision as BFT, the application of TOF in determination of body traits is promising. However, the animal effect is very large. Thus further analyses to specify the cows’ properties leading to the differences in image quality, reliability in measurement and trait values need to be carried out. For inferential statistics the cow effect should be taken into account, and additionally, more than one trait should be used as independent variable for estimating or predicting BFT or BCS. Larger datasets of camera traits should be prepared in order to analyze their usability in functional traits. To achieve a better quantification of the effect of the dairy cow’s breed, further studies should aspire a balanced design.

## Methods

The data used for analysis was collected from 96 HF cows from July 2011 to May 2012 at the dairy research farm Karkendamm of the Institute of Animal Breeding and Husbandry, Christian-Albrechts-University Kiel (Germany). Additionally, data was collected from 30 FV cows by the Institute for Agricultural Engineering and Animal Husbandry of Bavarian State Research Center for Agriculture in Grub (Germany) between August 2010 and July 2012. On both farms, prior recordings had taken place and were used for the control of the preprocessing results (paragraph "Control of preprocessing and determination of ROI").

### Manual gathering of BCS and BFT

In Kiel and Grub, BCS was determined within a 5 point scale (Wildman et al. 1982; Edmundson et al. 1989) with quarter point increments. On both participating farms, BFT was measured with a portable ultrasound generator (Tringa Linear VET, Esaote SPA, Italy). Within approximately one-quarter to one-fifth of the line connecting tuber coxae and tuber ischiadicum the point with maximal fat layer was chosen for measurement (Schröder and Staufenbiel 2006). As the linear distance between Kiel and Grub is ≈700 km, the BCS and BFT could not be gathered by the same personnel on both farms. In Karkendamm, an employee who was trained to measure BFT with the ultrasound generator gathered BFT on a weekly basis (N = 2848). In accordance with the farm’s usual operational procedures BCS (N = 710) was determined every four weeks by an experienced external BCS instructor. HF cows had a mean BFT of 9.71 mm (±3.78) and a mean BCS of 2.99 mm (±0.39). In Grub an employee gathered both BFT (N = 542) and BCS (N = 540) once a week at the day of TOF recording. The BFT measuring persons in Grub and Karkendamm had joint the same training on how to use the ultrasound generator. As a result of feeding experiments conducted in Grub, the herd’s range in body condition was above-average. FV cows had a mean BFT of 20.42 mm (±0.88) and a mean BCS of 3.76 mm (±0.43).

### Time-of-flight technology

The TOF camera (SR4000, Mesa Imaging AG) emits infrared light (modulated with 30 MHz), which is reflected by the object. The distance between object and camera is calculated from the phase shift. According to the manual, the camera’s range is 0.8 to 5 m, its accuracy of measurement is 1 cm, and it is recording up to 54 images (176 ×144 pixels) per second depending on the exposure time. The camera is able to output distance and (x, y, z) coordinate data, amplitudes, confidence maps as an estimate of reliability, and Swiss Ranger streams (srs) consisting of sequences of images (MESA-Imaging 2013). The cameras were provided by GEA.

### Installation and recording

In Karkendamm a camera was installed 2.55 m above one of four electronic feeding dispensers. The left wall was a solid stone wall of ≈3 m height and ≈2.5 m length. From this side no influence of sun light or other cows needed to be considered. The right wall is made of wood and only ≈0.3 m higher than the cows’ back. It did not stretch out of the cameras field of view. Cows could only enter or leave the box in a straight way or evade to the right. The camera was carefully positioned so that the visiting cow’s rear area was in the field of view while it was feeding (Figure 4). Recording was done with a 2-core system having 2 gb of RAM. Once the computer was switched on, recording started automatically ("Karkendamm.exe", Figure 1, paragraphs "Configuration and recording" to "Calibration and preprocessing"). Approximately every two weeks a TOF data recording was initiated and ran for averagely 4 days or until the removable hard disk was full. An RFID antenna (Nedap) near the feeder identified the cow by reading its responder. The eight-digit ID-number was gathered through serial communication by a splitter constructed by GEA.

In Grub a 2-core system having 3.43 gb RAM was used for short term recording once a week. The cows were individually led into a weighting box where top view recordings were taken from 2.55 m height. The used recording software ("Grub.exe") had a modified graphical user interface allowing the cows’ IDs to be entered manually. This was necessary, because no automatic ID system was present.

At both recording locations the SR4000 was used with default settings. After 1st of November 2011 only srs data was recorded.

### Dataset

The application "EKB" generated one list per recording day (subsection "Developed software") containing the output of "ROI" and "ExT" (paragraphs "Further processing: application "ROI"" and "Further processing: application "ExT""). These lists held values for every valid image of the recorded cows and had to be converted into a uniform dataset containing one value per trait, cow, and week.

In a first step, all values from one cow and recording day were within traits cleaned from outliers and afterwards averaged to produce one value per trait, cow, and day.

With this, the conversion of the FV dataset was complete, because recording and BFT -/BCS - determination had taken place at the same day once a week in Grub. The dataset contained at least 16 consecutive lactation weeks of each of the 30 considered FV cows.

As according to (Hady et al. 1994) the body condition scores determined in a 30 days interval are significant and precise, BCS had only been gathered every 4 weeks. The subsequent analysis of the camera traits’ changes indicated, that a reference measure with higher temporal resolution was required. Therefore, for Karkendamm only results related to BFT are presented. No influence could be taken which animal visited the electronic feeding dispenser at what time. Therefore, the days with camera traits did not coincide with the days of manually collected BFT values. In a second step, the Karkendamm data set had to be converted from a daily to a weekly basis, i.e. for every week the mean of all (daily based) camera traits’ values was taken to match the week’s BFT value. The final Karkendamm dataset included 96 dairy cows with at least 20 consecutive lactation weeks. None of these cows had more than one lactation period with at least 20 consecutive lactation weeks, thus only one lactation period per cow was considered.

### Additional information on the recorded dairy cows

Regarding the HF cows, 63 animals were in their first, 21 in their second, and 12 in their third to sixth lactation. When the recording period started, 32 cows were in the first lactation week, 35 cows were in the second to fifth lactation week, 24 cows were in the sixth to twelfth lactation week, and 5 cows were in lactation week 17 or higher (maximum of lactation weeks = 32, mean = 4.8, ± 5.4). The amount of consecutive lactation weeks in the dataset reached from 20 to 57 (mean = 35.3, ± 8.4). The milk yield ranged from 19.3 kg to 46.1 kg (mean = 34.1 kg, ± 4.9). The HF cows had a minimal and maximal body weight of 500.2 kg and 769.1 kg (mean = 626.1 kg, ± 64.9 kg), respectively.

Regarding the FV cows, 11 animals were in their first, 8 in their second, and 11 in their third to sixth lactation. At the beginning of the recording period, all cows were in their first lactation week. Some FV cows were needed for additional studies and taken out of the present study early. As only 6 cows have been recorded 20 or more consecutive lactation weeks, the minimum amount was lowered to 16. 24 cows have been recorded between 16 and 19 consecutive lactation weeks (max=26, mean=18.1, ±2.9). The milk yield ranged from 19.3 kg to 46.5 kg (mean = 32.9 kg, ± 7.5). The FV cows had a minimal and maximal body weight of 614.8 kg and 894.6 kg (mean = 741.6 kg, ± 67.9 kg), respectively.

### Descriptive statistics

For every trait sample size, minimum, maximum, mean value, standard deviation, and its correlation with BFT (and BCS for the Grub dataset) were calculated within breeds (Table 2).

To measure reliability in information gathering within cows, coefficients of determination were calculated for all traits restricted to each individual cow ( ). For both breeds has afterwards been separately tested, if weight, lactation number, lactation stage at the beginning of the recording period, or BFT/BCS starting level were significant for the -values.

In addition, coefficients of determination (*R*^2^) were determined for both breeds, to compare the overall precision in measurements between manually gathered traits and camera traits. Generalized linear models of the form *f*(*y*)=*b*∗*X* with a piecewise linear link function f and the trait’s observations of all cows were used. The predictor matrix X contained a constant term, lactation week, and the trait’s starting level as independent variables.

Additionally, the data was grouped within breeds with respect to cows and seasons, separately. Weeks 49 to 52 and 1 to 9 of each year belonged to season 1, weeks 10 to 22, weeks 23 to 35 and weeks 36 to 48 formed season 2, 3, and 4, respectively. Within performing the analyses of variance the effect sizes *η*^2^ were calculated in case significance was given.  is the proportion of variance in the data explained by the grouping. Comparisons of the means within the groups were plotted.

The descriptive statistics were calculated using MATLAB’s functions "mean", "std", "corrcoef", "anovan", "grpstats", and "glmfit" (MATLAB R2007a, ( The MathWorks I Statistics Toolbox For Use with MATLAB User‘s Guide)). As this study focused on feasibility and analyzing the measurement setting, inferential statistics have to be done in further investigations.

## Abbreviations

SR4000: SR4K: Swiss Ranger 4000, type designation of the 3D camera
BCS: Body condition score
BFT: Backfat thickness
TOF: Time of flight
HF (cow): Holstein Friesian (cow)
FV (cow): Fleckvieh (cow)
IMG: The latest image acquired from SR4000 while recording is running
SC-info: Scenery information
NUM: Number of images used to calculate SC-info
meanIMG: Image with pixelwise depth values’ means
stdIMG: Image with pixelwise depth values’ standard deviation
DUR: Duration until SC-info is renewed
ID-folder: Folder with recording data from one day belonging to one cow, named after the cow’s ID
RFID: Radio-frequency identification
(cow) ID: Animal identification according to RFID transponder
ROI: Region of interest (Figure 5)
hip2hip (HH): Area between the height profile connecting the cow’s hips and a horizontal line through the profile’s highest point
depthleft (DL): depthright (DR): Depth of the left/right dish of the rump (Figure 9)
volumeleft (VL): volumeright (VR): Cross sectional area of the left/right dish of the rump (Figure 9)
areapinleft (APL): areapinright (APR): Area enclosed by cow surface and the spatial line directly connecting hip and ischeal tuberosity on the left/right side of the cow’s back (Figure 9)
distpinleft (DPL): distpinleft (DPL): Distortion of the height profile connecting hip and ischeal tuberosity concerning the direct spatial line on the left/right side of the cow’s back (Figure 9)
areamidleft (AML): areamidright (AMR): Area enclosed by cow surface and the spatial line directly connecting hip and dish of the rump on the left/right side of the cow’s back (Figure 9)
distmidleft (DML): distmidright (DMR): Distortion of the height profile connecting hip and dish of the rump concerning the direct spatial line on the left/right side of the cow’s back (Figure 9)
fps: frames per second
SS: Sum of squares
*η*^2^: measure for the size of the effect of grouping data after a certain criterion, calculated from analysis of variance SS:
*R*^2^: Coefficient of determination; for all traits calculated from a generalized linear model using a piecewise linear link function
Coefficient of determination; for all traits calculated from a linear model within the cow’s data
GEA: GEA Farm Technologies, participating company
srs: Swiss ranger streams, data format of SR4000
gb: gigabyte
RAM: Random-access memory

## References

[CR1] Azzaro G, Caccamo M, Ferguson JD, Battiato S, Farinella GM, Guarnera GC, Puglisi G, Petriglieri R, Licitra G (2011). Objective estimation of body condition score by modeling cow body shape from digital images. J Dairy Sci.

[CR2] Bercovich A, Edan Y, Alcahantis V, Moallem U, Parmet Y, Honig H, Maltz E, Antler A, Halachmi I (2012). Automatic cow’s body condition scoring.

[CR3] Bewley JM, Peacock AM, Lewis O, Boyce RE, Roberts DJ, Coffey MP, Kenyon SJ, Schulz MM (2008). Potential for estimation of body condition scores in dairy cattle from digital images. J Dairy Sci.

[CR4] Brethour JR (1992). The repeatability and accuracy of ultrasound in measuring backfat of cattle. J AnimSci.

[CR5] Cohen J (1988). Statistical Power Analysis for the Behavioral Sciences.

[CR6] Collard BL, Boettcher PJ, Dekkers JC, Petitclerc D, Schaeffer LR (2000). Relationships between energy balance and health traits of dairy cattle in early lactation. J Dairy Sci.

[CR7] Crémer J (2013). A very minimal introduction to TikZ. Download: 1st Feb.

[CR8] Edmundson AJ, Lean IJ, Weaver LD, Farver T, Webster G (1989). A body condition scoring chart for holstein dairy cows. J Dairy Sci.

[CR9] Ferguson JD, Galligan DT, Thomsen N (1994). Principal Descriptors of Body Condition Score in Holstein Cows. J Dairy Sci.

[CR10] Ferguson JD, Azzaro G, Licitra G (2006). Body condition assessment using digital images. J Dairy Sci.

[CR11] Fietze S (2004). Vergleich der unterschiedlichen Konditionsbeurteilungsmethoden - Body Condition Scoring (BCS) und Rückenfettdickenmessung (RFD) - und deren Aussagefähigkeit in Bezug auf die Fruchtbarkeit von Holstein-Friesian (HF) Kühen. PhD thesis.

[CR12] (FNI) FI (2013). Informationsverarbeitung Sinnbilder für Datenfluß- und Programmablaufpläne, DIN 66001. Technical report, Deutschen Normenausschuß(DNA). Vieweg+Teubner Verlag.

[CR13] Hady PJ, Domecq JJ, Kaneene JB (1994). Frequency and precision of body condition scoring in dairy cattle. J Dairy Sci.

[CR14] Halachmi I, Klopcic M, Polak P, Roberts DJ, Bewley JM (2013). Automatic assessment of dairy cattle body condition score using thermal imaging. Comput Electron Agric.

[CR15] Hertem TV, Alchanatis V, Antler A, Maltz E, Halachmi I, Schlageter-Tello A, Lokhorst C, Viazzi S, Romanini CEB, Pluk A, Bahr C, Berckmans D (2013). Comparison of segmentation algorithms for cow contour extraction from natural barn background in side view images. Comput Electron Agric.

[CR16] Interbull (2012). Description of National Genetic Evaluation Systems for dairy cattle traits as applied in different Interbull member countries.

[CR17] Krukowski M (2009). Automatic Determination of Body Condition Score of Dairy Cows from 3D Images. Master’s thesis.

[CR18] MESA-Imaging (2013). SR4000 User Manual, version 2.0.

[CR19] Salau J, Junge W, Harms J, Suhr O (2011). Development and validation of an automatic optical system for the control of the body condition score of dairy cows.. Book of Abstracts of the 62th Annual Meeting of the European Federation of Animal Science.

[CR20] Schröder UJ, Staufenbiel R (2006). Methods to determine body fat reserves in the dairy cow with special regard to ultrasonographic measurement of backfat thickness. J Dairy Sci.

[CR21] The MathWorks I Statistics Toolbox For Use with MATLAB User‘s Guide Vers. 4 http://www.pi.ingv.it/~longo/CorsoMatlab/OriginalManuals/stats.pdf

[CR22] Weber A, Salau J, Haas JH, Junge W, Bauer U, Harms J, Bieletzki S, Suhr O, Schönrock K, Rothfuß H, Thaller G (2014). Estimation of backfat thickness using extracted traits from an automatic 3D optical system in lactating Holstein-Friesian cows. Livest Sci.

[CR23] Wildman EE, Jones GM, Wagner PE, Boman RL, Troutt HFJ, Lesch TN (1982). A dairy cow body condition scoring system and its relationship to selected production characteristics. J Dairy Sci.

